# Antimicrobial and Other Biomedical Properties of Extracts from *Plantago major*, *Plantaginaceae*

**DOI:** 10.3390/ph16081092

**Published:** 2023-08-01

**Authors:** Kairat Zhakipbekov, Aknur Turgumbayeva, Raushan Issayeva, Aliya Kipchakbayeva, Gulnara Kadyrbayeva, Meruyert Tleubayeva, Tamila Akhayeva, Kuanysh Tastambek, Gaukhar Sainova, Elmira Serikbayeva, Karakoz Tolenova, Balzhan Makhatova, Rabiga Anarbayeva, Zhanar Shimirova, Yerbol Tileuberdi

**Affiliations:** 1 School of Pharmacy, Asfendiyarov Kazakh National Medical University, Almaty 050000, Kazakhstan; zhakipbekov.k@kaznmu.kz (K.Z.); kadyrbaeva.g@kaznmu.kz (G.K.); meruert_iliasovna@mail.ru (M.T.); elmira.asyl@mail.ru (E.S.); makhatova.b@kaznmu.kz (B.M.); 2 Higher School of Medicine, Al-Farabi Kazakh National University, Almaty 050040, Kazakhstan; issayeva.raushan1@gmail.com (R.I.); akhaeva.tamila@kaznu.kz (T.A.); tastambeku@gmail.com (K.T.); tolenova.kd@gmail.com (K.T.); 3 Faculty of Chemistry and Chemical Technology, Al-Farabi Kazakh National University, Almaty 050040, Kazakhstan; aliya_k85@mail.ru; 4 Department of Biotechnology, M. Auezov South Kazakhstan University, Shymkent 160012, Kazakhstan; 5 Ecology Research Institute, Khoja Akhmet Yassawi International Kazakh-Turkish University, Turkistan 161200, Kazakhstan; bsainov@mail.ru; 6 Department of Drug Technology, South Kazakhstan Medical Academy, Shymkent 160001, Kazakhstan; rabiga.rm@mail.ru (R.A.); shimirova_z@mail.ru (Z.S.); 7 Institute of natural Sciences and Geography, Abai Kazakh National University, Almaty 050010, Kazakhstan; er.tileuberdi@gmail.com

**Keywords:** antimicrobial, antiviral, anti-inflammatory, wound healing, antigungal, *Plantago major*, extracts

## Abstract

Since ancient times, many scientists and doctors have used various herbs to treat diseases. Conventional drugs often have side effects, and pathogens are becoming resistant to these types of drugs. In such circumstances, the study of traditional medicinal plants is an effective and logical strategy for finding new herbal medicines. One such herb is *Plantago major*, a perennial plant in the *Plantaginaceae* family that is found throughout the world. The *Plantago major* plant has been used as a medicine for the treatment of various diseases. Studies have shown that plant extracts of *Plantago major* exhibit antimicrobial, antiviral, and anti-inflammatory effects, and have wound-healing properties. This review collects and presents the results of various studies of *Plantago major* plant extracts with antimicrobial, antiviral, antifungal, anti-inflammatory, and wound-healing properties, which demonstrate a wide range of therapeutic possibilities of *Plantago major* plant extracts and have a huge potential for use as a medicinal raw material.

## 1. Introduction

**Figure 1:**
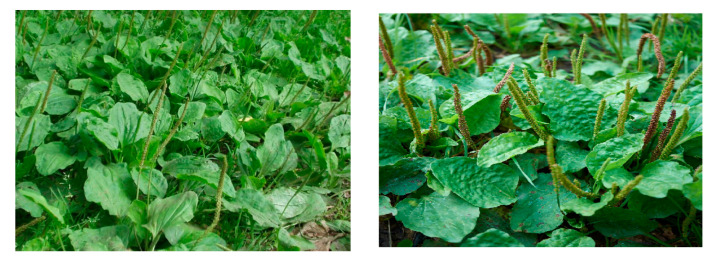
Picture of *Plantago major*.

**Figure 2:**
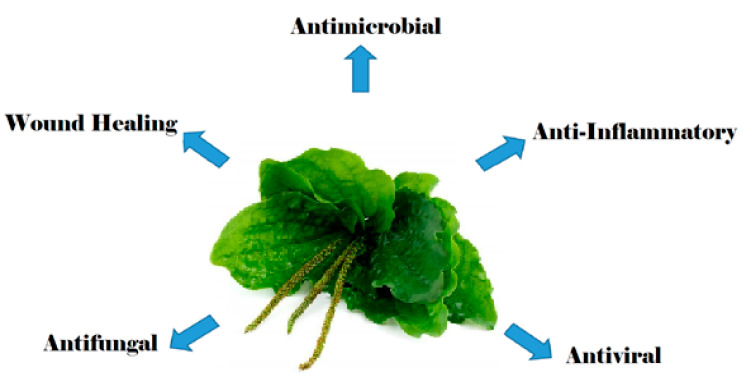
Pharmacological properties of *Plantago major*.

Plants have been used for their medicinal properties for thousands of years. Since ancient times, different types of herbs have been used by different scientists and physicians to treat diseases, and many books and treatises have been written describing the different effects of different plants on different diseases [1,2]. *Plantago major* is one of these herbs whose value has been praised since time immemorial [3]. *Plantago major* (*Plantago major* ssp. *Major* L.) is a perennial plant belonging to the *Plantagina’ceae* family (Figure 1) [4]. *Plantago major* was once distributed mainly in Europe and North and Central Asia, and is now widely distributed throughout the world [5,6,7]. *Plantago major* has been used to treat a variety of diseases, including infectious diseases [8,9]. It is reported that *Plantago major* has antimicrobial, antidiabetic, antispasmodic, antiviral, anti-inflammatory, and wound-healing properties (Figure 2) [10,11,12]. The emergence of drug-resistant pathogens in recent decades has prompted scientists to evaluate the effects of medicinal plants on pathogens [13]. The study of plants traditionally used as medicines should still be seen as a useful and logical research strategy in the search for new antimicrobials and other anti-infectives [14]. Conventional chemicals have numerous side effects compared to their herbal counterparts [15,16]. Plant extracts are an attractive alternative as they are a rich source of biodegradable secondary metabolites such as phenols, flavonoids and saponins, alkaloids, etc., which have antimicrobial, anti-inflammatory, wound-healing and antiviral properties and reduce the likelihood of disease [17,18,19,20]. The main purpose of this review is to introduce the wide therapeutic potential of *Plantago major* extracts and the possibility of their use as herbal medicines.

## 2. Methods

We searched the literature on scientific search engines such as Scopus, Clarivate, MDPI, Wiley Online PubMed, ScienceDirect, and Google Scholar from 1990 to 2022 to study *Plantago major* extracts as well as its pharmacological properties such as antimicrobial, anti-inflammatory, wound-healing, antiviral and antifungal properties. *Plantago major*, *Plantago major* extracts, antimicrobial properties of *Plantago major*, anti-inflammatory properties of *Plantago major*, wound-healing properties of *Plantago major*, antiviral properties of *Plantago major*, and “medical use” were used as keywords.

## 3. Phytoconstitutions

**Figure 3:**
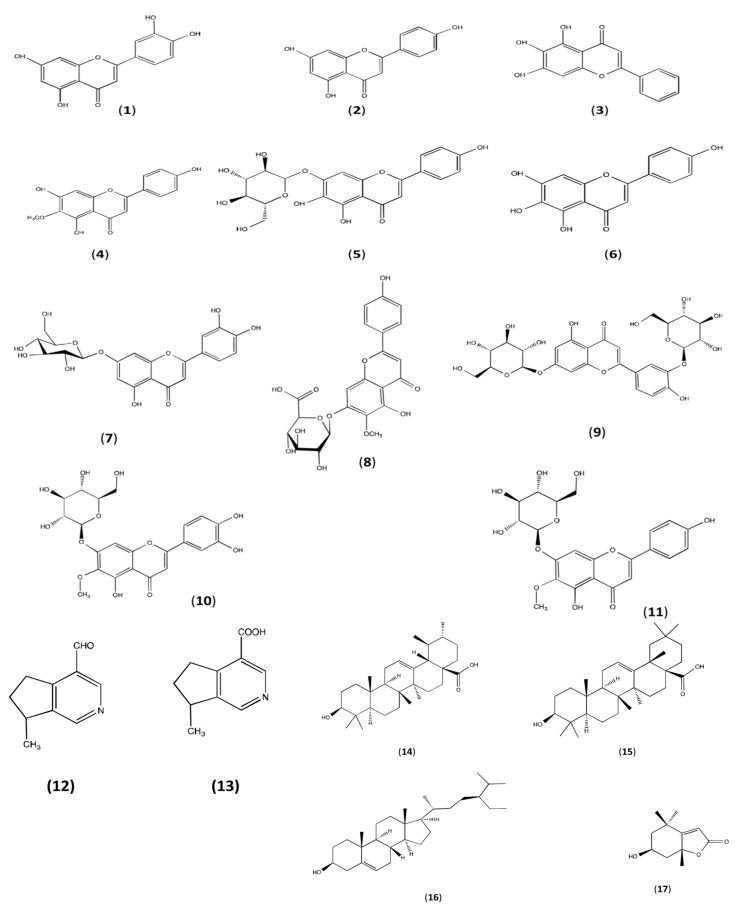

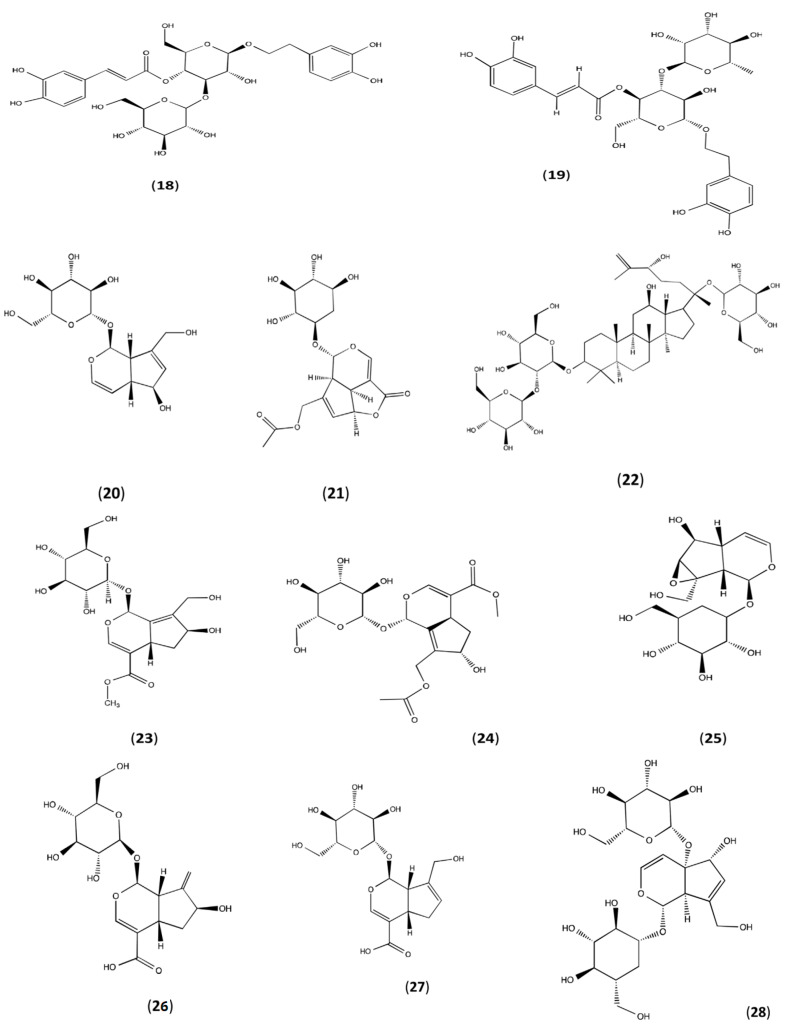

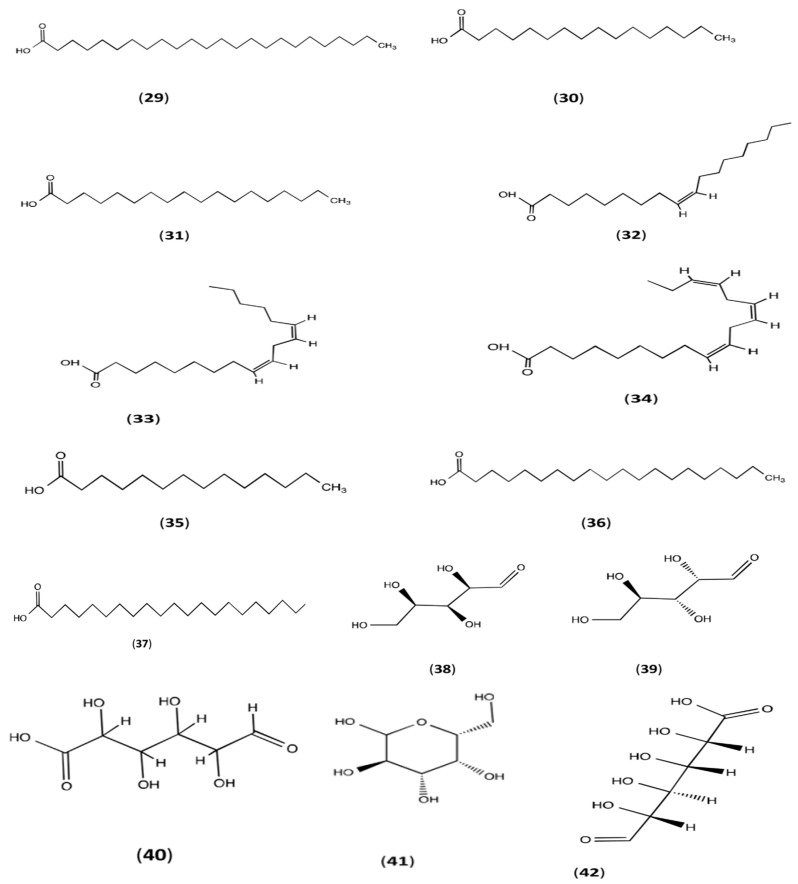

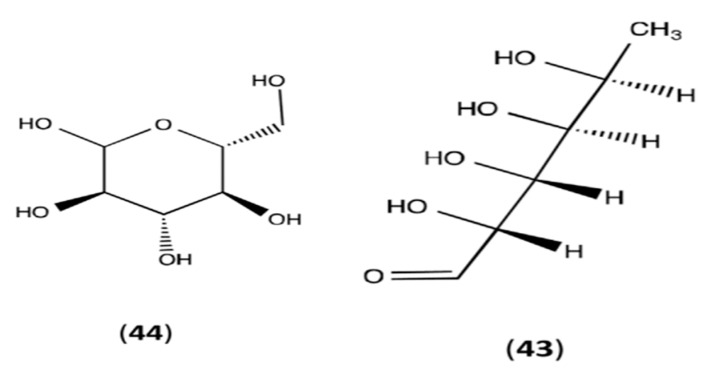
Chemical structure of biologically active substances of *Plantago major*.

*Plantago major* is a significant medicinal plant that includes a wide range of bio-active substances, including phenolic compounds, flavonoids, terpenoids, iridoid glycosides, alkaloids, fatty acids, and polysaccharides (Figure 3). The seeds, leaves, flowers, and roots of the plant, as well as practically every other part of it, contain these compounds [21]. 

Flavonoids are present in *Plantago major*, which is widely known. The principal flavonoids found in it are flavones such luteolin (**1**) and apigenin (**2**). The flavonoids baicalein (**3**), hispidulin (**4**), plantagin (**5**), and scutellarin (**6**) have been isolated from this plant. *Plantago major* from Egypt was used to isolate the flavonoids luteolin 7-glucoside (**7**), hispidulin 7-glucuronide (**8**), luteolin 7-diglucoside (**9**), apigenin 7-glucoside (**10**), nepetin 7-glucoside, and luteolin 6-hydroxy 4′-methoxy 7-galactoside. Homoplantaginin (**11**) was just recently found. The presence and quantity of flavonoids in *Plantago major* methanol extracts were confirmed by LC-MS/MS. Aucubin, baicalein, leuteolin, and baicalin, the glucuronide of baicalein, were the flavonoids that were extracted from the aqueous extract [21,22,23,24,25,26,27,28]. 

The alkaloids indicain (**12**) and plantagonin (**13**) of *Plantago major* were also isolated [21,29]. 

*Plantago major*’s wax and leaves have terpenoids in them. The leaves contained loliolid (**17**), while the leaf wax extract in 95% ethanol included ursolic acid (**14**), oleanolic acid (**15**), sitosterol acid (**16**), and 18-glycyrrhetinic. The same terpenoids were found in hexane extract as well [21,30,31,32,33].

The caffeic acid derivatives plantamajoside (**18**) and acteoside (**19**), often referred to as verbacoside, were discovered in *Plantago major*. Additionally, it was discovered that the methanol extract contains more plantamajoside than acteoside. However, the amount of plantamajoside in the 80% ethanol extract is comparable to the amount of acteoside [21,34]. 

The important iridoid glycoside aucubin (**20**) was isolated from the leaves of *Plantago major*. From many different plant sections, several iridoid glycosides have been discovered. Numerous studies have shown that *Plantago major*’s aerial parts contain iridoid glycosides, and asperuloside (**21**) was isolated from the flowers. These include the substances found in *Plantago major*’s aerial portion, such as majoroside (**22**), 10-hydroxymajoroside (**23**), and 10-acetoxymajoroside (**24**), as well as catapol (**25**), gardoside (**26**), geniposidic acid (**27**), and melittoside (**28**) [21,35,36,37,38,39].

The seeds and leaves of *Plantago major* have also been used to separate fatty acids. Lignoceric acid was extracted from the seeds (**29**). Using gas–liquid chromatography, permanganate oxidation, and spectrophotometric techniques, it was also discovered that *Plantago major* contains the following acids: palmitic acid (**30**), stearic acid (**31**) oleic acid (**32**) linoleic acid (**33**) and linolenic acid (**34**) as well as others. Myristic acid was additionally extracted from the seeds (**35**). A minor naturally occurring component of fatty acids in *Plantago major* seed oil extracted from gasoline is 9-hydroxy-cis-11-octadecenoic acid. Behenic acid (**37**) and arachidic acid (**36**) were also extracted from the leaves [21,40,41,42,43]. 

Polysaccharides are present in *Plantago major* seeds. Also isolated were xylose (**38**), arabinose (**39**), galacturonic acid (**40**), and galactose (**41**), which was discovered in a hot water extract. Additionally, glucuronic acid (**42**), rhamnose (**43**), galactose, and glucose (**44**) were separated from a 50 °C water extract [21,44,45,46,47,48,49].

## 4. Antimicrobial Effects

Plantamajoside and phenyl propanoid glycoside, which are derived from *Plantago major* and represent 3,4-dihydroxy-phenethyl-O-d-glucopyranosyl-(13)-4-O-caffeoyl-d-glucopyranoside, demonstrated the value of the minimum inhibitory concentration for seven phytopathogenic bacteria. Following preliminary agar diffusion testing, *Escherichia coli* (30 mg/mL) and *Staphylococcus aureus* (50 mg/mL) showed the best performance [50]. 

Research on the antibacterial efficacy of extracts against *Pseudomonas aeruginosa* isolated from burn infections shows that different concentrations of the ethanol extract of *Plantago major* leaves (100, 75, 50, 25, and 10%) have different zones of inhibition against *P. aeruginosa* with a diameter ranging from 9.93 mm to 22.18 mm. With rising extract concentration, the zone of inhibition grew larger. With a 22.18 mm diameter zone, a 100% alcohol extract showed the strongest inhibitory effect. This bacterium has shown a high level of antibiotic resistance [51]. A bioinformatic approach was used in the study by Abbasi et al. (2022) to assess the antibacterial activity of *Plantago major* extracts against *Pseudomonas aeruginosa* as well as their capacity to affect the expression of the Tox-A gene. The results showed that the raw extracts had good antibacterial activity when compared to other fractionated plant extracts. *Plantago major* has an average zone of inhibition of up to 16 mm against *P. aeruginosa* at 1000 mg/mL. In comparison to the aqueous extract, which displayed an inhibition zone, petroleum ether, and chloroform had a minor inhibitory effect. The Tox-A gene expression value was significantly (*p* < 0.05) reduced at most plant concentrations [52]. 

The antimicrobial activity of extracts from dried *Plantago major* leaves at a concentration of 50 mg/mL each, obtained by two different extraction techniques, ultrasonic (40 kHz) (US) and classical (maceration) (CE), was tested on two species of Gram-positive bacteria (*Staphylococcus aureus, Bacillus subtilis*), Gram-negative bacteria (*Pseudomonas aeruginosa*), two yeasts (*Candida albicans, Saccharomyces cerevisiae*) and one mold (*Aspergillus niger*). The methanol extract did not inhibit any of the test organisms. Regardless of the extraction method used, hydroalcoholic extracts of *Plantago major* leaves showed superior antibacterial activity against yeast compared to both Gram-positive and Gram-negative bacteria. Recovery methods have been shown in situations involving *Staphylococcus aureus* and *Pseudomonas aeruginosa*. *Escherichia coli* was more easily defeated by the extract made with UE, whereas *Bacillus subtilis* and both yeasts were more easily defeated by the extract made with CE (95 percentile of confidence). *Saccharomyces cerevisiae* was the most sensitive organism, with a diameter of 19.2 ± 0.5 and 23.5 ± 0.1 mm for extracts obtained by CE and UE, respectively. The extract produced by classical extraction had a higher overall concentration of phenolic compounds and flavonoids than the extract produced by ultrasonic extraction even though ultrasound had a positive impact on the yield of extractives and the kinetics of extraction [53]. However, in vivo tests of antimicrobial activity and the minimum effective dose of hexane, methanol, ethanol, and an aqueous extract of *Plantago major* leaves by Akkuş and Hiziçekliyurt (2021) revealed that all extraction techniques of *Plantago major* leaves have activity against *Bacillus subtilis, Staphylococcus aureus* and *Pseudomonas aeruginosa, Candida tropicalis*, and *Candida albicans* at 4 mg/mL. Extracts of *Plantago major* in methanol and ethanol were more effective than those in hexane and water. The lowest activity (8 mg/mL) against the bacteria *Escherichia coli* and *Pseudomonas vulgaris* was found in the *Plantago major* hexane and aqueous extract. The antibacterial properties of *Plantago major* ethanol and methanol extracts were effective against *Staphylococcus aureus, Enterococcus faecalis,* and *Pseudomonas aerouginosa* at 2 mg/mL and 4 mg/mL, respectively [54]. Additionally, an ethanolic extract of *Plantago major* leaves is effective in inhibiting the growth of *Streptococcus pyogenes* ATCC 19615 at dosages of 250 mg/mL, 500 mg/mL, 750 mg/mL, and 1000 mg/mL [55]. Furthermore, methanolic extracts of *Plantago major* leaves showed higher antibacterial activity than aqueous and alcoholic extracts in studies conducted by Soliman et al. (2022). A methanolic extract of *Plantago major* leaves from canal banks displayed the highest efficacy against *P. aeruginosa*, followed by an extract from orchards against *Staphylococcus aureus* [56]. *Plantago major* leaf extracts can inhibit microorganisms because they contain flavonoid components, phenols, tannins, and terpenoids, which all function together synergistically. Through the peptidoglycan of bacteria, chemicals can enter the cell and influence the cytoplasmic membrane. Due to the leakage of cytosolic fluid, structural changes in the membrane occur, including fluidity alterations and changes in the outer layer of the cell wall. This is the first step in the inhibitory process for phenolic, flavonoid, and isoflavonoid compounds. This leak induced morphological alterations in the bacterial cells, including death [57].

Three common pathogens, *Staphylococcus aureus, Escherichia coli,* and *Listeria monocytogenes*, are all susceptible to the antibacterial effects of *Plantago major* seed aqueous extracts. The MIC of the aqueous extract ranges from 0.09 to 0.181 mg/mL for *E. coli* and *L. monocytogenes* and from 0.022 to 0.045 mg/mL for *S. aureus*. The strains were entirely resistant to the alcoholic seed extract in this investigation as well, hence it is still unknown what the MICs and MBCs of the strains are [58]. However, an alcohol extract of *Plantago major* seeds gathered in Semnan Province (Iran) was found to have antibacterial activity in a study by Mirkalantari and Fateh (2018) [59]. The lowest concentration of *Plantago major*’s alcoholic extract, which inhibits *Staphylococcus aureus* growth, was 100 mg/mL with an inhibition diameter of 13 mm. However, the acetone extract of *Plantago major* seeds had a stronger antibacterial effect than its alcoholic extract, and it showed an antibacterial effect on *Streptococcus mutans* at a concentration of 1 mg/mL with an average diameter of the growth inhibition zone of 6 ± 0.2 mm. This was demonstrated by the antibacterial properties of *Plantago major* seed extracts in vitro against *S. mutans* at eight different concentrations of acetone and ethanol. This extract had an MBC and MIC of 1 mg/mL and 0.5 mg/mL, respectively. At a dosage of 0.5 mg/mL and an average inhibition zone diameter of 8.3 ± 0.1 mm, the alcoholic extract of *Plantago major* seeds inhibited the growth of *S. mutans*. This extract had an MBC and MIC of 8 mg/mL and 2 mg/mL, respectively. At greater doses of both extracts, growth in the diameter of the zone of inhibition was seen. The chlorhexidine (CHC) growth inhibition zone had an average diameter of 24 mm [60]. The antimicrobial effect of an aqueous–alcoholic extract of *Plantago major* (a native strain of Khorasan, Iran) on *Streptococcus mutans* also showed good antimicrobial activity and an average growth inhibition zone diameter of 15 ± 1 mm. At a dosage of 1.56 mg/mL, a large seed extract containing zinc oxide nanoparticles exhibited antibacterial action. The average inhibitory zone diameter for the same extract at the same concentration but without zinc oxide nanoparticles was 7.67± 0.57 mm (Table 1) [61]. In another study of the crude extract and aqueous phase of the seeds of the plant *Plantago major* growing in the territory of Turkey, it was found that the crude extract and the aqueous phase of *Plantago major* have a weak antimicrobial effect on *S. aureus, Proteus mirabilis, S. pyogenes*, and *E. coli*, compared with the highest bacteriostatic and bactericidal effect on individual populations of bacteria of the ethyl acetate fraction. During the study, the total content of phenols and flavonoids was found in the composition, which may be the cause of activity [62]. *Plantago major* demonstrated a MIC for *S. aureus* and *E. coli* of 6.25 mg/mL and 25 mg/mL, respectively, in a broth microdilution test [63]. *Plantago major* seeds include phenolic compounds, tannins, and flavonoids that provide seed extracts with a strong antibacterial effect. These compounds have the antibacterial qualities of inhibiting the synthesis of DNA and RNA since the B ring of flavonoids makes hydrogen bonds with the bases of nucleic acids to prevent the formation of new nucleic acids. The findings of decreased fluidity of cell membranes in the presence of flavonoids support the idea that flavonoids have an inhibitory effect on how the cytoplasmic membrane operates. Another way that flavonoids work to inhibit bacterial growth is by blocking energy metabolism. By disrupting energy metabolism, lipidchalcones may affect how much energy is required for metabolite uptake or the synthesis of macromolecules. Another secondary active metabolite, phenol, prevents bacterial development because of its toxicity. The amount of hydroxyl groups attached to the phenol group controls how harmful phenols are to microorganisms since higher hydroxylation results in increased toxicity. Microbe toxicity may be brought on by non-specific interactions of phenols with proteins or by the inhibition of enzymes brought on by the reaction of oxidized compounds with sulfhydryl groups. Tannin is a class of polyphenolic chemicals that binds to various organic materials like proteins and amino acids via covalent, hydrogen, and hydrophobic interactions. Thus, tannin’s ability to inactivate microbial adhesins and transport proteins or enzymes accounts for its antibacterial properties [64].

Against the bacterial strains *Escherichia coli* VKPM-M17, *Salmonella typhimurium* TA 100, and *Staphylococcus aureus* MDC 5233, an ethanolic extract of *Plantago major* root exhibits modest antibacterial activity. Although all strains of bacteria, including *Escherichia coli* VKPM-M17, *Salmonella typhimurium* TA 100, and *Staphylococcus aureus* MDC 5233, are susceptible to the acetone extract of *Plantago major* roots, it is nonetheless effective at different concentrations. Acetone extract performed well when used against *Bacillus cereus* (3.562 mg/mL), while performing poorly when used against *B. subtilis, P. mirabilis, P. aeruginosa,* and *S. epidermidis* (28.500 mg/mL) [65]. However, investigations by Metiner et al. (2012) showed that all the bacteria utilized in the study—aside from *E. coli* and *B. cereus*—are resistant to the ethanol extract of *Plantago major*’s roots [66]. At the same time, two strains of *Porphyromonas gingivalis* and *Fusobacterium nucleatum* in vitro were inhibited by ethanol extracts of the root of *Plantago major* at doses of 75% and 100%. In contrast to 70° alcohol, where bacterial inhibition was not seen, inhibition halos averaged 14.9 mm when 0.12% chlorhexidine +0.05% cetylpyridinium chloride was used [67]. Additionally, the evaluation of the antibacterial activity of *Plantago major*’s root methanol extracts at various concentrations (1000, 500, 250, and 125 mg/mL) on six different bacterial strains, including *Lactobacillus* sp., *Staphylococcus aureus*, *Proteus* sp., *Pseudomonas aeruginosa, Escherichia coli* and *Enterococcus* sp., revealed a zone of inhibition within 25 ± 1.3 and 10 ± 0.6 mm (Table 1) [68]. The biological activity and therapeutic advantages of *Plantago major* roots are influenced by the characteristics of the chemically active constituents. Polysaccharides and polyphenols may have biological effects, according to some research. However, it is not apparent how precisely bacteria are suppressed, or which bio-active compound is most crucial.

The *Plantago major* extract demonstrated a good inhibitory ability against primary plaque colonizers and periodontal bacteria, according to an in vitro study of its antimicrobial activity. *Plantago major* has maximal mean ZOI values of 9.2 ± 1.09 mm and 10.6 ± 0.54 mm, respectively, at 24 h, suggesting possible antibacterial action against periodontal pathogens and primary plaque colonizers [69]. Also, agar disk diffusion research on hydroalcoholic *Plantago major* extracts revealed antibacterial activity against five oral bacterial strains: *Fusobacterium nucleatum* ATCC 25586, *Actinomyces viscosus* ATCC 15987, *Streptococcus mutans* ATCC 25175, *Lactobacillus acidophilus* ATCC 314, and *Prevotella melaninogenica* ATCC 25845. In comparison to ethyl alcohol (5.8 mM) and Perio Aid^®^ (chlorhexidine 0.012%) (22.0 mM), hydroalcoholic extracts at both concentrations (25 g/mL and 50 g/mL) displayed greater antibacterial activity. The hydroalcoholic extract of *Plantago major* at a concentration of 25 g/mL had the maximum activity. Etanolic and hydroalcogolic *Plantago major* extracts contain mucilages, pectins, flavonoids, tannins, and glycosides like aucubin and catalpol among their active ingredients. The active component aucubigenin, which is generated from aucubin, is crucial because, during catabolism, it creates a dialdehyde that acts as a bactericide by denaturing the proteins of oral bacteria and plaque colonizers. It also has various flavonoids, of which acteoside and plantamajoside have antibacterial properties [70].

In the course of studying the antimicrobial activity and microbiological purity of CO_2_ extracts, it was found that the extract exhibits antimicrobial activity against cultures of Gram-positive bacteria, as well as antifungal activity against *Candida albicans* [71]. Green synthesis of silver nanoparticles (Ag NPs) using *Plantago major* plant extracts also shows promising antibacterial activity against *Staphylococcus aureus* and *E. coli* bacteria at a dosage of 20 µg/mL [72]. A very crucial antibacterial mechanism is oxidative stress. Ag NPs can generate superoxide radicals, reactive oxygen species (ROS), and hydroxyl ions, all of which can quickly destroy microorganisms. The disruption of the cell membrane, penetration of the cell membrane, and binding to DNA and protein result in ROS. Ag NPs’ antibacterial activity is most likely caused by their surface charge, which attracts negatively charged bacteria [73].

An alternate hope in the struggle against bacteria that are resistant to antibiotics may be *Plantago major*. According to the findings of the research conducted by Arslan et al. (2018), the extract demonstrated a synergistic effect against *Acinetobacter baumannii* with amoxicillin/clavulanic acid and ceftriaxone, and the zone of inhibition was 28 mm against this microorganism [74].

## 5. Antiviral and Antifungal Effects

Evaluation of the antiviral activity of the aqueous extract and pure compounds of the *Plantago major* against several viruses, namely herpesviruses (HSV-1, HSV-2) and adenoviruses (ADV-3, ADV-8, ADV-11) showed that the aqueous extract of the *Plantago major* has only slight activity against the herpes virus. On the contrary, some pure compounds belonging to five different classes of chemicals found in the extracts of this plant showed potent antiviral activity. Among them, caffeic acid showed the highest activity against HSV-1 (EC_50_ = 15.3 µg/mL, SI = 671), HSV-2 (EC_50_ = 87.3 µg/mL, SI = 118), and ADV-3 (EC_50_ = 14.2 μg/mL, SI = 727), while chlorogenic acid had the strongest activity against ADV-11 (EC_50_ = 13.3 μg/mL, SI = 301). The pure basic compounds of *Planatgo major* with antiviral activity are mainly derived from phenolic compounds, especially caffeic acid. Its mechanism of action against HSV-2 and ADV-3 has been found to be at the multiplication stage (HSV-1 post-infection: 0–12 h; ADV-3: 0–2 h) and at SI values greater than 400 [75]. In another study, Chiang et al. (2003) also tested the antiviral activity of *Plantago major* hot water extracts in vitro against herpes viruses (HSV-1 and HSV-2), adenoviruses (ADV-3, ADV-8, and ADV-11). The results showed that the hot water extract of *Plantago major* had a significant inhibitory activity also against viral infection (HSV-2 and ADV-11), which confirms the previous results and the high antiviral activity of *Plantago major*. The low antiviral activity of the aqueous extract of *Plantago major* can be explained by the low concentration of phenolic compounds present in the extract. The results on the time effect between 0 and 12 h after infection with the virus showed that caffeic acid tends to inhibit the replication of the virus. This suggests that the mode of action is not due to inhibition of viral absorption, but due to viral replication after infection. Phenolic compounds exhibit the best antiviral and antiadenoviral activity of the five chemical classes studied. Contrary to substances with one hydroxyl group at the R1 position, such as ferulic acid and p-coumaric acid, substances with two hydroxyl groups at the R1 and R2 positions of the cinnamic acid fragment, such as caffeic acid and chlorogenic acid, displayed a wider spectrum of antiviral activity. Thus, the spectrum of antiviral activity of phenolic compounds containing a cinnamic acid fragment is associated with the number of hydroxyl groups in positions R1 and R2. The property of direct antiviral action, strengthening of cellular immunity, and secretion of interferon can explain the reason for the antiviral activity of the *Plantago major* [76].

Verbascoside, a phenylpropanoid glycoside isolated from *Planatago major* seeds, affects the morphological development of pathogenic fungi at low concentrations. Verbascoside inhibited in vitro growth of *Botrytis cinerea, Fusarium culmorum*, and *Bipolaris sorokiniana*, but not *Alternaria solani* and *Rhizoctonia solani* [77]. High-concentration alcoholic extracts of plantain 7.5, 15, and 30 mg/mL. have antifungal properties. In the presence of 30 mg/mL, the diameter of the growth halo increased to 12.4 mm. As for the MIC, MIC50, and MIC90 indicators, they are 38, 14, and 29 µg/mL, respectively. Taking into account the number of living cells, the smallest number of living cells was formed when taking 10 mg/mL. The average value of this indicator was obtained for *Planatago major* 4.2 CFU/mL [78]. The alcoholic extract of *Plantago major* is also involved in the fight against fungal infections within a group of fungal strains. Maximum growth inhibition (32.2%) was obtained against *P. cinnamomi* at 2000 µg/mL followed by *C. gloeosporioides* (25.7%) on Day 6 and *C. godetiae* and *C. nymphaeae* (21.1%) on Day 9 [79].

## 6. Anti-Inflammatory Activities

*Plantago major* leaf, extracted with chloroform, n-hexane, methanol, and their respective extracts, inhibited ear dermatitis caused by Croton oil in mice. Each extract (300 μg/cm^2^) caused a significant reduction in edema, the most active extract with chloroform. Its effectiveness was only two times lower than that of the reference drug indomethacin: their ID_50_ values (the dose that provides 50% edema suppression) were 177 and 93 µg/cm^2^, respectively [80]. In addition, water and ethanol-based extracts of *Plantago major* leaves have an anti-inflammatory effect on oral epithelial cells in vitro. Concentration 0.1 mg/mL (with total phenols content of 1.67, 0.22, and 0.94 mg and content of 1.24, 0.19, and 0.71 mg GAE plantamaoside per ml for ethanolic, aqueous, and combination of both extracts, respectively) exhibited the highest anti-inflammatory activity against oral epithelial cells (OEC H400). The anti-inflammatory response was reduced or absent at both the lower and higher concentrations tested. The fact that the total phenol content and plantamajoside content of the ethanol-based extract at 0.01 mg/mL (0.12 mg plantamajoside and total phenol content of 0.17 mg GAE per mL) is comparable to the plantamajoside and total phenol content of the extracts water-based at 0.1 mg/mL, but there is a significant difference between the anti-inflammatory response of both extracts in vitro, indicating that not only total phenols and plantamajoside but also other water-soluble components (possibly polysaccharides) are responsible for the ultimate wound-healing and anti-inflammatory effects of *Plantago major*. However, both the ethanol-based and mixed forms of the water- and ethanol-based extracts demonstrated anti-inflammatory action at a concentration of 1.0 mg/mL [81].

In another study, treatment of inflammation with *Plantago major* leaf in rats with acetic acid-induced ulcerative colitis showed positive results. The leaf extract at high doses significantly reduced ulcerative index and histopathological lesions, as well as tissue levels of IL-6, TNF-α, PGE2, IL-1β, MPO, and MDA compared to the lesion group. The leaf extract at low doses also significantly reduced the levels of several markers [82]. Along with this, *Plantago major* leaf extracts attenuate inflammatory activity in the inflammatory response following acetaminophen (APAP) hepatotoxicity. The anti-inflammatory activity of ethanol, aqueous, and methanol extracts in vitro was 12.23 ± 3.15%, 26.74 ± 1.6%, and 21.69 ± 2.81%, respectively. In comparison to the treated groups, the APAP group had considerably higher levels of pro-inflammatory cytokines (IL-1, and TNF-α). Before leukocyte migration into tissues, IL-1 and TNF-α that are generated by active macrophages stimulate leukocyte adhesion to endothelial surfaces. TNF-α and IL-1 frequently express synergistic action to initiate cell death. The plasma levels of TNF-α and IL-1 indicate how severe the inflammation is. The partial avoidance of APAP toxicity demonstrated by the suppression of TNF- and IL-1 includes a decrease in the amount of liver enzyme in circulation. The TNF-α and IL-1 levels were lower in the *Plantago major* treated groups. The methanol extract treated group saw a more pronounced impact, which was consistent with *Plantago major*’s in vitro anti-inflammatory properties. It indicated that the anti-inflammatory property of the *Plantago major* was able to prevent the inflammatory reaction following APAP toxicity. It showed that the anti-inflammatory ability of *Plantago major* might stop the inflammatory response brought on by APAP poisoning. The liver contains 11-HSD type 1, which is responsible for converting inactive glucocorticoids into their active form. The nuclear transcription factors AP-1 and NF-B, which promote the production of all pro-inflammatory cytokines, are suppressed by the anti-inflammatory effects of glucocorticoids. The *Plantago major*’*s* methanol and ethanol extract treated groups revealed increased activity and expression of the 11-HSD type 1 enzyme. As a result, local tissue production of active glucocorticoids may attenuate the inflammatory response [83].

The anti-inflammatory effect of the *Plantago major* seed extract at various doses (100, 300, 1000 mg/kg) was compared with sodium salicylate (300 mg/kg) and distilled water when administered intraperitoneally. To investigate this effect, 2.5% formalin was subcutaneously injected into the paws of male rats. Plethysmometry was used to measure inflammation on the first day (acute inflammation) and within seven days (chronic inflammation). There was no discernible difference between sodium salicylate at a dose of 300 mg/kg (*p* < 0.003) and *Plantago major* extract at a dose of 1000 mg/kg (*p* < 0.003) when it came to their acute anti-inflammatory effects. At 1000 mg/kg, the *Plantago major* extract considerably reduced chronic inflammation on Days 4, 7, and 8 (*p* < 0.05); however, sodium salicylate showed that effect only on Day 4, and other extracts had no impact [84].

A microemulsified ethanolic extract of *Plantago major* had an anti-inflammatory effect on Croton oil-induced ear edema in mice. Local application of microemulsions reduced ear edema and the number of pro-inflammatory cells in the tissue, and its activity was similar to that of 1% hydrocortisone (*p* > 0.05) [85]. Aqueous and ethanolic extracts of *Plantago major* have an anti-inflammatory effect at doses of 20 and 25 mg/kg. It was established that the decrease in inflammation was 90.01% when using indomethacin as a control marker, and *Plantago major* 3.10% when using 5 mg/kg, 41.56% when using 10 mg/kg, 45.87% when using 20 mg/kg and 49.76% when using 25 mg/kg. The mean effective dose (ED_50_) of *Plantago major* was 7.507 mg/kg [86]. In addition, pretreatment with an aqueous extract (1 g/kg, po) of *Plantago major* reduced acetic acid-induced writhing in mice, but did not alter the tail-flick response to thermal nociceptive stimuli. In rats, an aqueous extract (1 g/kg, po) reduced carrageenan-induced paw edema and pleurisy but did not eliminate dextran-induced paw edema. The effect of the aqueous extract on carrageenan inflammatory responses was more intense than on-ear edema induced by Croton oil in mice. In addition, daily administration of an aqueous extract (1 g/kg/day for 8 days, orally) inhibited the exudative process induced by the administration of Croton oil into the rat air sacs. Aqueous and ethanolic extracts of *Plantago major* contain a large amount of biologically active compounds such as terpenoids and fatty acids, as well as some of their structural derivatives. Some of the naturally occurring fatty acids and triterpenoids, as well as all the semi-synthetic thioester-containing fatty acids, inhibit COX-2 catalyzed prostaglandin biosynthesis, where alpha-MNCs are sensitive to COX-2 suppression. As you know, COX-2 is a source of prostanoids that are formed during inflammation. The anti-inflammatory effect of *Plantago major* is largely associated with its inhibitory effect on COX-2 [87].

**Figure 4:**
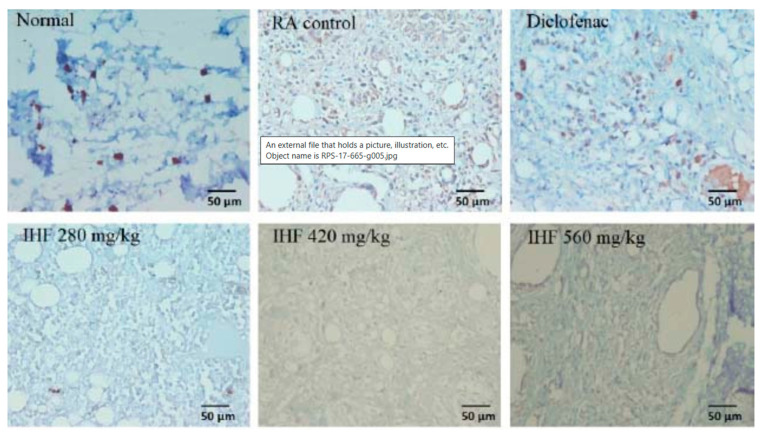
Histopathological data of CFA-induced suppression of RA with IHF. In five different fields, the histopathological features of joint tissue were magnified 400 times (scale bar: 50 m). Representative images of cyclooxygenase 2-expressing cells (dark brown stained) in the paw edema. RA, Rheumatoid arthritis; IHF, n-hexane-insoluble fraction. 1. Normal rat paw (no rheumatoid arthritis, **upper left**) 2. Rat paw with rheumatoid arthritis (**upper center**) 3. Effect of diclofenac on the paw of a rat with rheumatoid arthritis (**upper right**) 4. Effect of IHF at 280 mg/kg on the paw of a rat with rheumatoid arthritis (**lower left**) 5. Effect of IHF at 420 mg/kg on the paw of a rat with rheumatoid arthritis (**lower center**) 6. Effect of IHF at 560 mg/kg on the paw of a rat with rheumatoid arthritis (**lower right**).

Soluble (SHF) and insoluble (IHF) dichloromethane extract (DCM) fractions of *Plantago major* fractionated with n-hexane had anti-inflammatory properties in three animal models with paw edema. DCM, SHF, and IHF decreased leukocyte migration in mice and prevented paw edema in rats (Figure 4). The proportion of inhibitory effect for DCM, IHF, and SHF at a dose of 560 mg/kg was 47.33%, 55.51%, and 46.61%, respectively. IHF at doses of 280 and 560 mg/kg decreased osteoclast development and COX-2 expression in an animal model of RA when compared to diclofenac (Table 2). This activity is reported to be associated with several compounds, namely oleic acid, linoleic acid, palmitic acid, and oleamide, identified in DCM, IHF, and SHF. Through processes that do not need peroxisome proliferator-activated receptor gamma (PPAR), palmitoleic acid improves insulin sensitivity and lowers hepatic inflammation in rats. Nuclear factor (NF) B is inhibited when the PPAR is activated, which lowers the expression of pro-inflammatory genes and the generation of cytokines such as tumor necrosis factor-alpha (TNF-α) and interleukin (IL)-12. Additionally, it has been demonstrated that oleic and linoleic acids reduce inflammation in rat macrophages by controlling the production of inflammatory mediators including IL-6, IL-1, and cytokine-induced neutrophil chemoattractant-2αβ. DCM, IHF, and SHF inhibited leukocyte migration in a model of thioglycolate-induced peritonitis (Figure 4). This is consistent with the results of several previous studies that examined the activity of oleic acid in the peritoneal lavage of mice suffering from sepsis. Oleic acid increased the levels of the anti-inflammatory cytokines IL-10 and decreased the levels of the pro-inflammatory cytokines TNF-α and IL-1β. Similarly, oleic acid inhibited lipopolysaccharide-induced cell migration in isolated human neutrophils. In an animal model of CFA-induced rheumatoid arthritis, IHF reduced the onset of arthritis and COX-2 expression. This action is associated with the activity of oleic and linoleic acids as inhibitors of COX-2 [88].

## 7. Wound-Healing Effects

NO inhibitor assay, fibroblast proliferation assay, and high glucose migration assay have been used to study the effect of in vivo wound healing and in vitro wound healing in patients with diabetes, and to monitor potential skin irritation after topical application of *Planatgo major* leaf extracts, whose main biologically active compounds are ursolic acid (UA) and oleanolic acid (OA), in hyperglycemic rats. These studies have shown that UA can promote this wound-healing process with PMLE, UA, and OA did not cause significant skin irritation when applied topically. PMLE and UA have anti-inflammatory properties as they significantly reduce NO formation in RAW264.7 cells following LPS administration at all doses. In addition, NO generation was reduced by OA (2.5–10 g/mL). Fibroblast proliferation is one of the important phases of tissue regeneration. NIH/3T3 fibroblast cells were used to study the concentration range of PMLE, UA, and OA during the proliferation phase. PMLE at 31–500 μg/mL promoted fibroblast proliferation, although UA and OA at 7.81–125 μg/mL had the opposite effect [89]. We also evaluated the clinical efficacy of a hydroalcoholic extract of *Plantago major* in the healing of diabetic foot ulcers (DNS) and bedsores (NS) by applying a 10% gel with an extract of *Plantago major*. The use of *Plantago major* extract gel for topical application to the wound once per day along with dressing and standard wound care for two weeks demonstrated a larger reduction in wound size compared to the control after the first week (64.90 ± 29.75% vs. 33.11 ± 26.55%; *p* < 0.001) and the second week (86.85 ± 24.34% vs. 52.87 ± 32.41%; *p* < 0.001). Additionally, the proportion of patients in the medication group (*n* = 32, 64%) who had fully healed their wounds was higher than that of the control group (*n* = 9, 20.45%; OR: 3.129, 95% CI: 1.685–5.80; *p* < 0.001). This effect is due to the polyphenolic compounds and the antioxidant properties of the extract. The polyphenolic compound in the *Plantago major* extract, called plantamajoside, is anti-inflammatory and is responsible for the extract’s healing properties. It has also been noted that proteins isolated from the plantain extract affect the proliferation of fibroblasts, which leads to accelerated wound healing. Nitric oxide (NO) generation is raised by *Plantago major* extract, and this effect may aid in the healing of wounds. Hispidulin and baicalein, two of *Plantago major’s* primary flavonoids, exhibit anti-inflammatory and antifungal effects. Additionally, they prevent the release of histamine and prostaglandins, preventing their tissue-damaging effects. Additionally, the astringent action of flavonoids may play a key role in speeding up wound healing [90].

The effect of a 50% aqueous solution of *Plantago major* on the healing of burn wounds was compared with 20% silver sulfadiazine and ozerin in rats. There was no significant difference between the groups of rats given these drugs on Days 7 and 14, but the difference was significant on Day 21. The best results were noted in the group treated with 50% of the *Plantago major* [91].

In a study by Zubair et al. (2016) the wound-healing properties of *Plantago major* extracts (water and ethanol) were evaluated using an ex vivo wound-healing model in pigs. Both types of extracts stimulated wound healing in porcine skin, but the ethanol-based extracts were somewhat more effective. A concentration of 1.0 mg/mL (based on dry weight) gave the best results for both extract types. The fact that ethanol-based polyphenol-rich extracts stimulated wound-healing activity to a larger extent than water-based extracts with comparable amounts of dried *Plantago major* leaves suggests that polyphenol content has a substantial impact. The considerably favorable effects of the water-based extracts at doses of 1.0 and 0.1 mg/mL point to some water-soluble chemicals in these extracts having wound-healing characteristics. Several substances may work alone or in concert, or substances that stimulate or control other substances during wound healing may be involved. Plantamajoside, acteoside, aucubin, and ursolic acid are among the chemicals that are present in *Plantago major* leaves and have wound-healing properties [92]. In a study on the effect and toxicity of ethanolic extracts of *Plantago major* on wound healing compared to a commercial product used in Brazil, macroscopic and microscopic analysis showed a good level of wound healing. After trauma to the cervicodorsal region of mice, *Plantago major* extract and ointment were applied to the cervicodorsal region of mice. The reduction in wound area occurred earlier in mice treated with *Plantago major* than in the control marker, ointment. On the 15th day, wounds treated with *Plantago major* had complete wound closure. Microscopic analysis of the wound on the ninth day revealed that animals treated with *Plantago major* had more effectively formed neoepithelium and skin appendages, whereas, during ointment therapy, reepithelialization and the absence of skin appendages in the lesion were absent. Wound-healing activity is associated with a high content of steroids and tripertenes and not only. Triterpenoids inhibit cyclooxygenase-1 and -2, which catalyzes the biosynthesis of prostaglandins in vitro, while the structural isomer of oleanolic acid is less active. However, the wound-healing potential is the result of a combination of phytocomponents and the real contribution of each of them. Coumarins caused significant changes in the regulation of immune responses. Alkaloids helped to improve the healing of incisions by increasing the tensile strength of scar tissue. In addition, it enhanced wound healing, accelerating the period of epithelialization and contraction of the wound on the 4th and 16th days. In addition, dose-dependently accelerates the recovery of soft tissues in the initial phases of the process due to chemotactic properties in relation to fibroblasts [93].

Nanostructured electrospun dressing PCL with an aqueous ethanolic extract from the leaves of *Plantago major* encapsulated in nanofibers, developed for use in wound healing, showed a minimum inhibitory concentration (MIC) of 5.3 mg/mL for methicillin-resistant *Staphylococcus aureus* [94]. In an in vitro scratch assay, *Plantago major* leaf extracts based on water and ethanol promoted cell proliferation and migration. They also showed anti-inflammatory effects in an in vitro NF-kB assay using oral epithelial cell cultures. Similar to ex vivo testing employing detached pig ears, these extracts promoted wound healing [95]. Along with this, the ethanolic extract of *Plantago major* leaves and roots has a wound-healing effect on rats with hyperglycemia. The results of the study show that the gel of alcoholic extract of *Plantago major* leaves and roots at a concentration of 5% and 10% can increase the percentage of wound closure and accelerate wound-healing time (*p* < 0.05) [96]. Even though there was no significant difference on Day 0 (*p* = 0.69), there was a significant difference in the average wound surface area on Day 14 (*p* = 0.014) in another study of the effect of a methanol extract of the leaves of *Plantago major* on the duration of wound healing in male rats [97]. Antioxidant substances such as plantaginin, baicalein, and hispidulin, which are found in *Plantago major*, guard cells against being destroyed by inflammatory mediators. The migration of cells and proliferation are crucial to the healing of wounds. After treatment with *Plantago major* leaf extract, bio-active compounds such as polyphenols and polysaccharides encourage cell proliferation and migration and enhance wound healing.

Evaluation of the impact on Wistar rats’ local wound healing of an ointment made from a combination of cabbage, pomegranate, and *Plantago major* ethanolic extract found that on Days 3, 6, 9, and 12, the average percentage of wound healing in the various groups was as follows: In the control group, there were 21.3%, 37.87%, 67.39%, and 77.17%; in the positive control group, there were 24.98%, 34.21%, 70.74%, and 88.55%; and in the ointment group, there were 32.35%, 61.27%, 94.53%, and 99.91%. There was a noticeable difference between the control and main groups (*p* < 0.05) [98]. The topical application of 10% *Plantago major* leaf extract (PMLE) to infected wounds in rabbits confirmed that PMLE plays a role in promoting healing. These results were in parallel with the WCR results, which reflect the mean wound contraction rate at Days 7, 14, and 21 in the PMLE-treated group was significantly higher (*p* < 0.001) than in the normal saline-treated group. The results of histopathological examination confirmed an early increase in new blood vessel formation, fibroblast proliferation, marked collagen precipitation, and early epithelialization in the PMLE group compared to the control group [99]. A mixture of *Plantago major* and aloe vera on the process of wound healing in rat models according to stereological parameters also showed a good wound-healing effect when applied topically. The group of rats treated with a gel containing 5% *Plantago major* and 5% blend of aloe vera (PA group) showed a faster rate of wound closure compared to the control group and the gel base group (*p* < 0.05). The number density of fibroblasts, the volume density of collagen bundles, and the average diameter and volume density of vessels in the PA group were significantly higher than in the control group and the group that received the gel base (*p* < 0.05). Overall, the topical efficacy of *Plantago major* in skin wound healing in animal models in terms of wound contraction rate was observed at 10%, 20%, and 50% concentrations compared with A mixture of *Plantago major* and aloe vera on the process of wound healing in rat models according to stereological parameters also showed a good wound-healing effect when applied topically. The group of rats treated with a gel containing 5% *Plantago major* and 5% blend of aloe vera (PA group) showed a faster rate of wound closure compared to the control group and the gel base group (*p* < 0.05). The number density of fibroblasts, the volume density of collagen bundles, and the average diameter and volume density of vessels in the PA group were significantly higher than in the control group and the group that received the gel base (*p* < 0.05). Overall, the topical efficacy of *Plantago major* in skin wound healing in animal models, in terms of wound contraction rate, was observed at 10%, 20%, and 50% concentrations compared with control preparation ozemecine [100]. Topical application of *Plantago major* extracts accelerates the healing of infected wounds due to the content of phenols and flavonoids, which act as antioxidants and anti-inflammatory substances, enhancing WCR due to early and additional fibroblast proliferation and angiogenesis compared with the control. group. This improvement in wound healing is due to two main factors. First, *Plantago major* extracts contain phenolic compounds with antiseptic and antioxidant properties in addition to flavonoid compounds with antioxidant and anti-inflammatory properties. Second, due to the high percentage of the use of *Plantago major* extracts (5–10%), which, as it turned out, is the reason for the acceleration of the wound-healing process. On the other hand, *Plantago major* extracts reduce bacterial growth by stimulating fibroblast proliferation. The use of a dressing also contributes to the acceleration of the wound, since the dressing promotes faster epithelialization, and collagen synthesis, promotes angiogenesis, creates hypoxia in the wound bed, and reduces the pH of the wound bed, which leads to a decrease in wound infection [101].

## 8. Conclusions

This paper presents studies of *Plantago major* extracts carried out carefully and in full. The results of the study, performed superficially and together with other plant studies, were not included in this review. The results presented in the review give a clear idea of the therapeutic possibilities of the plant *Plantago major*. Various pharmacological effects of *Plantago major* extracts, including antibacterial, anti-inflammatory, wound-healing, antiviral, and antifungal have been demonstrated by a lot of studies that are now available. The therapeutic properties of the plant are due to its numerous chemical components. Phenolic chemicals are regarded as the most significant and active compounds among the numerous substances present in *Plantago major* plant essence. The bio-active compounds found in *Plantago major* are used in the manufacturing of a variety of drugs and are crucial to maintaining human health. The most well-known and effective research has been done on *Plantago major’s* antibacterial, anti-inflammatory, and antiviral in numerous animal models. Future research should therefore focus on the potential use of this plant for the treatment of many non-communicable disorders.

## Figures and Tables

**Table 1:** Minimum Inhibitory Concentration of *Plantago major* extracts.

No.	Extraction	Part of Plant	Minimum Inhibitory Concentration (MIC) (mg/mL)										
			Bc *	Bs *	Sa *	Se *	Ec *	Kp *	Pa *	Pm *	Sen *	P. g *	F. n *
1	Aceton extract	Leaf, seed, root	3.5	8.	4.25	28.5	14.2	14.2	28.5	8.25	7.13		
2	Ethanol extract	Leaf, seed, root	42.5	R	R	R	42.5	R	R	R	R	-	0.93
3	AgNO_3_	unknown	6.85	-	5.7	-	9.5	-	6.8	-	-	-	-
4	Diethyl ether extract	Leaf, seed	-	-	-	-	5.83	-	-	-	-	-	-
5	Water extract	Leaf, seed	-	-	-	-	-	-	125	-	-	-	-
6	Hydroalcoholic extract	Leaf, seed	-	-	8.8	-	-	-		-	-	-	11.0
7	Ultrasonic Extract	Leaf	-	10.7	10.7	-	11.7	-	11.3	-	-	-	-
8	Classical Solvent Extract	Leaf	-	11.0	11.3	-	10.9	-	11.2	-	-	-	-
9	Ethyl acetate extract	Leaf	33.5	-	16.7	-	-	67	33.5	67	-	-	-
10	Aqueous extract	Leaf, seed	-	-	57.2	-	-	-	114.5	-	-	-	
11	Ag/AgCl nanoparticles	unknown	-	-	0.8		1.6	-	-	-	-	-	-
12	Alcoholic extract	Leaf, seed	-	-	0.04	-	0.03	-	-	-	-	-	-
13	CO_2_ extract	unknown	-	18	18	-	16	-	-	-	-	-	-
14	Zinc oxide nanoparticles	Seed	-	-	1.56	-	-	-	-	-	-	-	-

* Se: Staphylococcus epidermidis, Pa: Pseudomonas aeruginosa, Sa: Staphylococcus aureus, Bc: Bacillus cereus, Pm: Proteus mirabilis, Kp: Klebsiella pneumonia, Bs: Bacillus subtilis, Ec: Escherichia coli, F. n: F. nucleatum, P. g: P. gingivalis, Sen: Salmonella enteritidis, R: Resistant.

**Table 2:** Investigation methods of extracts from *Plantago major*.

No.	Pharmacological Activity	Extraction	Part of Plant	Concentration		Pre-Clinical Method
				mg/mL	%	
1	Antimicrobial	Aceton extract	Leaf, seed, root	500	-	in vivo
		Ethanolic extract	Leaf, seed, root	100, 75, 50, 25	10%, 75%, 100%	in vivo
		AgNO3	unknown	20	-	in vivo
		Diethyl ether extract	Leaf, seed	0.016–1.0	-	in vivo
		Hydroalcoholic extract	Leaf, seed	25	-	in vivo
		Ultrasonic Extract	Leaf	50	-	in vivo
		Metanolic Extract	Leaf, seed, root	1000, 500, 250, 125	-	in vivo
		Ethyl acetate extract	Leaf	0.078–5.0	-	in vivo
		Aqueous extract	Leaf, seed	20, 25	-	in vivo
		Alcoholic extract	Leaf, seed	25, 50, 75, 100, 1000	10%, 75%, 100%	in vivo
		CO_2_-extract	unknown	18	-	in vivo
		Zinc oxide nanoparticles	Seed	1.56	-	in vivo
2	Anti-Inflammatory	Ethanolic extract	Leaf	0.1	-	in vitro
		Water extract	Leaf	0.1	-	in vitro
		Metanolic Extract	Seed, leaf	50	-	in vitro
		n-hexane	Seed, leaf	280, 560	-	in vitro
		Chloroform	Seed, leaf	280, 560	-	in vitro
		DCM	Seed, leaf	280, 560	-	in vitro
3	Wound Healing	Hydroalcoholic extract	Leaf, root	-	50%	in vitro
		Aqueous extract	Leaf	1.0	-	in vitro/ex vivo
		Ethanol extract	Leaf, root	1.0	-	in vitro/ex vivo
		Salve	Leaf	-	10%	in vitro
4	Antiviral	Aqueous extract	Leaf	14.2; 15.3; 87.3	-	in vivo
5	Antifungal	Alcoholic extract	Seed, leaf	7.5, 15, 30	-	in vivo
		Ethanol extract	Seed	2000	-	in vivo

## Data Availability

Not applicable.
